# An International System of Electro-Therapeutics, for Students, General Practitioners and Specialists

**Published:** 1894-08

**Authors:** 


### Book Reviews.

---

An International System of Electro-Therapeutics, for Students, General Practitioners and Specialists. By Horatio R. Bigelow, M.D., and thirty-eight associate editors. Thoroughly illustrated. In one large royal octavo volume, 1,160 pages, extra cloth, $6.00 net: sheep, $7.50 net; half Russia, $7.50 net. Philadelphia: The F. A. Davis Co., Publishers, 1914 and 1916 Cherry street.

It is hardly possible, in the space allowed the reviewer, to do justice to such a work. The editor, with the aid of thirty-eight associates, has succeeded in giving to the profession, in one volume, a large amount of most valuable matter, both to the physician and to the student. The book is arranged systematically, giving first facts, of which both physicians and students are generally ignorant, in electro-physics, animal electricity, static electricity and magnetism, galvanism, electro-physiology and diagnosis, etc. The application of the different forms of electricity to various diseased organs and disordered functions is then taken up by the different editors, whose names alone are a sufficient guarantee of the correctness of their conclusions. No physician of to-day is fully equipped unless he possess a fair knowledge of the therapeutic applications of electricity, and we know of no work better suited to his needs than the above.

